# Relationship between shipping amounts of olive flounder aquacultured from Jejudo and the reported events of acute food poisoning by *Kudoa septempunctata* in 2015, South Korea: an ecological study

**DOI:** 10.4178/epih.e2017041

**Published:** 2017-09-06

**Authors:** Jong-Myon Bae

**Affiliations:** Department of Preventive Medicine, Jeju National University Scool of Medicine, Jeju, Korea

**Keywords:** Virulence, Food parasitology, Parasitic intestinal diseases, Myxozoa, Ecological bias

## Abstract

**OBJECTIVES:**

Confirmation of *Kudoa septempunctata* (*K. septempunctata*) as the pathogenic agent causing acute food poisoning remains under debate owing to inconsistencies in the reproducibility of experimental evidence. Higher intake of olive flounder infected with *K. septempunctata* would result in increased diagnosis of food poisoning by *K. septempunctata*, if the latter was one of the causal agents of acute food poisoning. The aim was to evaluate the relationship between the shipping amount of olive flounder aquacultured from Jejudo and the incidence of *K. septempunctata* food poisoning in 2015, Korea.

**METHODS:**

Data of shipping amounts between March 2014 and February 2016 and of monthly reported events of *Kudoa* food poisoning were taken from Jejudo Fish-Culture Fisheries Cooperatives and Korea Centers for Disease Control and Prevention, respectively. Non-parametric correlation analyses were conducted.

**RESULTS:**

Shipping amounts indicated the seasonal changes according to variation of consumption. Spearman’s rho and Kendall’s tau-a between the monthly shipping amounts and the reported events in 2015 were 0.39 (p=0.21) and 0.27 (p=0.20), respectively.

**CONCLUSIONS:**

An independent relationship was noted between the shipping amount and the reported events, which contrasted with the claim that the virulence of *K. septempunctata* caused acute food poisoning.

## INTRODUCTION

In December 2012, it was first claimed internationally that *Kudoa septempunctata* (*K. septempunctata*) found in some of the cultured olive flounders aquacultured in Jejudo, South Korea (hereafter Korea) [1], and had caused food poisoning [2,3]. Subsequently, the Korea Centers for Disease Control and Prevention (KCDC) reported 11 events occurred in 2015 [4]. In that report, the *K. septempunctata* food poisoning was defined as “those where the 18S ribosomal RNA gene of *K. septempunctata* had been detected in the vomit or excreta of patients with acute food poisoning symptoms who reported consuming sliced raw fish of olive flounders.”

The virulence of *K. septempunctata* was first noticed from experiments using 4-5 days old Deutschland, Denken, and Yoken mice [2]; however, other researchers were unable to reproduce this pathogenesis upon conducting the same experiments [5-7]. As such, the causality of *K. septempunctata* food poisoning has remained controversial due to irreproducibility of study results [8]. Moreover, it was suggested that the 11 events of food poisoning that reported in 2015 might not have been caused by *K. septempunctata*. First, because epidemiologic characteristics, including incubation period and clinical symptoms suggested that the food poisoning could be caused in combination with either *Staphylococcus aureus* or *Bacillus cereus*; especially because of the difficulty in detecting toxins and pathogenic agents for both types of food poisoning during the epidemiological investigations [9]. Second, owing to the differences in the monthly distribution of the 11 events. About 75% of *K. septempunctata* food poisoning in Japan occurred between August and November [3], whereas a total of 4 events had reported in Korea during the same period with a proportion of 36.3%, showing only half compared to Japan [4]. In addition, 4 events reported only during May in Korea, while there was no event in June and July, so that the validity of the definition for *K. septempunctata* food poisoning made by KCDC seemed doubtful.

On the other hand, as there were differences in reported events depending on the month, correlation with monthly variation of sales amounts of olive flounders aquacultured from Jejudo needed to be considered [9]. In other words, if *K. septempunctata* were the pathogenic myxozoa causing the acute food poisoning, there would be a higher risk of food poisoning events when the intake of olive flounder aquacultured from Jejudo increased. Thus, this study aimed to investigate the correlation between the monthly distribution of the reported events of *K. septempunctata* food poisoning reported by the KCDC in 2015, and the monthly shipping amounts of olive flounder that Jejudo Fish-Culture Fisheries Cooperatives (http://www.jaf-suhyup.co.kr/, hereafter JFC) supplied to the Korean domestic markets during the same period. It was expected that the present ecological study would add evidence to evaluate the hypothesis that *K. septempunctata* was the pathogenic myxozoa causing acute food poisoning.

## MATERIALS AND METHODS

The JFC kindly provided the monthly shipping data for olive flounders aquacultured from Jejudo between March 2014 and February 2016 (Appendix 1). To investigate the trend of seasonal shipping amounts, the data were reclassified into March-May, June-August, September-November, and December-February in the next year.

Information about *K. septempunctata* events was based on the 2015 report on *K. septempunctata* food poisoning published by the KCDC in 2016 [4]. These 11 events summarized by month were the first reported Korean events where the KCDC interpreted national *K. septempunctata* food poisoning reports.

To investigate the correlation between shipping amounts during each month of 2015 and *K. septempunctata* food poisoning events during same time, coefficients for non-arametric methods, Spearman‘s rho and Kendall’s tau-a, were calculated with a pvaule=0.05 significance level. To evaluate the delay effect, crosscorrelation of time series analysis was calculated; considering that there was 12 months of data, a 3 months time delay was set. These analyses were conducted by using Stata version 14 (StataCorp., Collage Station, TX, USA).

## RESULTS

**Figure 1.**
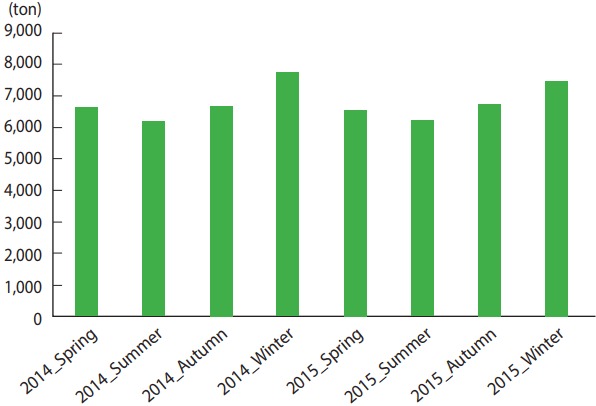
Seasonal shipping amounts of olive flounder from Jejudo between Spring 2014 and Winter 2015.

**Figure 2.**
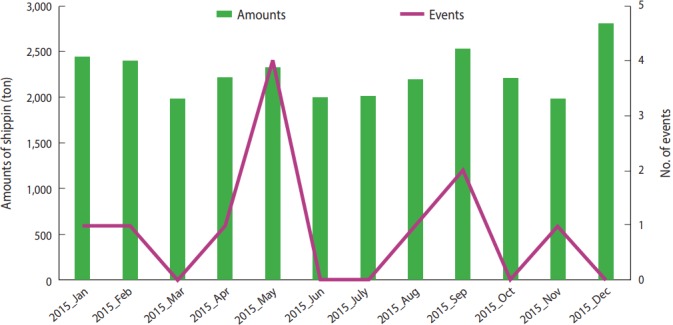
Seasonal shipping amounts of olive flounder from Jejudo and incidence of K. septempunctata food poisoning in 2015, Korea.

When seasonal shipping amounts by JFC in 2014 - 2015 were checked, there were seasonal changes in the shipping amount in the order of winter-fall–spring–summer (Figure 1). In 2015, when monthly shipping amounts and the events by month were considered together, there was no event in December when the shipping amount was highest, while there was 1 event in November when there was lowest shipping amount (Figure 2). In addition, May had the highest number of events with the fifth highest shipping amount. All non-parametric correlation analyses between shipping amount and number of events found that they were independent with a p= 0.05 (Spearman’s rho= 0.39, p= 0.21; Kendall’s tau-a = 0.27, p= 0.20). In addition, cross-correlation found no correlation depending on delay by month (Table 1).

## DISCUSSION

The present study identified that monthly shipping amounts of olive flounders aquacultured in Jejudo in 2015, and the events of *K. septempunctata* food poisoning in the same month were mutually independent. The results of this ecological study had two meanings. First, *K. septempunctata* food poisoning as defined by the KCDC needs to be reviewed. Second, accepting the present definition would be supporting the evidence that *K. septempunctata* is not a pathogenic myxozoa causing acute food poisoning in humans, but just a parasite in olive flounders.

The KCDC interpreted that the monthly variation of reported events was due to the difference in detection rate depending on sea water temperature [4]. However, it was claimed that *K. septempunctata* was detected throughout the year regardless of sea water temperature [10]. Furthermore, it was also reported that *K. septempunctata* was not detected in wild olive flounders [11]. Therefore, it seems unlikely that the seasonal variation in events was attributable to the difference in sea water temperature.

On the other hand, most events occurred during the summer and the fall in Japan [3], whereas there was no event during the summer (July and August) and the highest was in spring (May) in Korea [4]. Therefore, it would be more reasonable to interpret the difference based on the variation of chance for the intake of sliced raw olive flounders by month, instead of change in water temperature. The present study also found that seasonal difference was not attributable to the variation of shipping amounts.

The present study had four major limitations. First, there may be differences between the shipping amount of olive flounders used for analyses in this study and the actual amounts consumed. However, cultured olive flounders to be used for sliced raw fish needed to be delivered alive to the ultimate consumers, so shipping amounts were determined by amounts ordered. Thus, it can be considered that shipping amounts and consumed amounts should be directly proportional, albeit not exactly the same. This was overcome by correlation analyses. Second, if there were time gaps between shipping and intake, there would be no correlation. However, olive flounders were mostly consumed as sliced raw fish, so in restaurants they tend to be consumed as soon as possible in order to be provided in the freshest state. While the time gap may be a few days, it hardly goes beyond a month. In addition, the present study was analyzed by month, not by day, and the cross-correlation analysis confirmed that there were no variations dependent on delay duration. Third, this study used the number of events for *K. septempunctata* food poisoning from the report by the KCDC, while numbers of patients per each event were not used. *K. septempunctata* food poisoning was caused by a common cause [4], in which there were various numbers of patients per event. Thus, it was regarded more reasonable to use number of events for ecological analysis rather than number of patients. Fourth, this study was designed as an ecological analysis, in which ecologic fallacy by using group data, instead of individual data, could be introduced [12]. Thus, the results of this study should not claim that causality was revealed, but instead, should propose a new hypothesis [13]. While previous studies claimed that *K. septempunctata* could be pathogenic myxozoa causing acute food poisoning, the present study suggested that epidemiologic study should be conducted to find a more valid causality.

## Figures and Tables

**Table 1.** Cross-correlation coefficients by time lags

Time lag (mo)	Cross-correlation coefficient
-3	0.000
-2	0.077
-1	0.000
0	0.237
1	0.000
2	-0.007
3	0.000

## Appendix 1. Shipping amounts of olive flounder from Jejudo from January 2014 to February 2016

**Table:**

Time	Amount (ton)
2014_Jan	1,858
2014_Feb	2,038
2014_Mar	2,262
2014_Apr	2,143
2014_May	2,225
2014_Jun	2,197
2014_July	2,066
2014_Aug	1,922
2014_Sep	2,099
2014_Oct	2,564
2014_Nov	2,002
2014_Dec	2,906
2015_Jan	2,443
2015_Feb	2403
2015_Mar	1,986
2015_Apr	2,220
2015_May	2,335
2015_Jun	2,000
2015_July	2,013
2015_Aug	2,199
2015_Sep	2,531
2015_Oct	2,214
2015_Nov	1,988
2015_Dec	2,809
2016_Jan	2,290
2016_Feb	2,371
