# Supplementary material for: Relationship between shipping amounts of olive flounder aquacultured from Jejudo and the reported events of acute food poisoning by Kudoa septempunctata in 2015, South Korea: an ecological study
Source: Epidemiol Health. 2017 Sep 6;39:e2017041. doi: 10.4178/epih.e2017041 (PMC5675978; doi:10.4178/epih.e2017041)
Supplement: Supplementary file 1 [file epih-39-e2017041-supplementary.pdf]

<Brief Communication>

2015년도 제주도 양식 광어 출하량과 쿠도아충 발생사례: 생태학적 연구

Relationship between Shipping Amounts of Olive Flounder Aquacultured from Jejudo and the Reported Events of Acute Food Poisoning by ***Kudoa septempunctata*** in 2015, South Korea : An Ecological Study

Abstract

Objectives: Confirmation of *Kudoa septempunctata* as the pathogenic agent causing acute food poisoning remains under debate owing to inconsistencies in the reproducibility of experimental evidence. Higher intake of olive flounder infected with *K. septempunctata* would result in increased diagnosis of food poisoning by *K*. septempunctata, if the latter was one of the causal agents of acute food poisoning. The aim was to evaluate the relationship between the shipping amount of olive flounder aquacultured from Jejudo and the incidence of *K. septempunctata* food poisoning in 2015, Korea.

Methods: Data of shipping amounts between March 2014 and February 2016 and of monthly reported events of Kudoa food poisoning were taken from Jejudo Fish-Culture Fisheries Cooperatives and Korean Centers for Disease Control and Prevention, respectively. Non-parametric correlation analyses were conducted.

Results: Shipping amounts indicated the seasonal changes according to variation of consumption. Spearman's rho and Kendall's tau-a between the monthly shipping amounts and the reported events in 2015 were 0.39 (p-value=0.21) and 0.27 (p-value=0.20), respectively.

Conclusions: An independent relationship was noted between the shipping amount and the reported events, which contrasted with the claim that the virulence of *K. septempunctata* caused acute food poisoning.

Keyword: Virulence, Food parasitology, Parasitic intestinal diseases, Myxozoa, Ecological bias

서론

한국 제주도에서 양식하여 국내외로 출하되는 광어 (olive flounder)의 일부에서 발견되는 *Kudoa septempunctata* (이하 칠성쿠도아충) [1]이 식중독을 일으킨다는 주장이 2012년 12월에 전세계적으로 처음 제기되었다 [2,3]. 이에 따라 한국의 질병관리본부는 2015년도에 발생한 쿠도아 식중독 (Kudoa food poisoning) 11건 사례를 발표하였다 [4]. 이 보고서에서 쿠도아 식중독 사례를 '급성 식중독 증상을 보이면서 광어회 섭취력을 가진 사례에 대하여 토사물 혹은 배설물에서 쿠도아충 18S ribosomal RNA gene (rDNA)이 검출된 경우'로 정의하고 있다.

그러나 칠성 쿠도아충이 병인성을 갖는다는 처음으로 주장한 근거는 출생 4-5일된 Deutschland, Denken, and Yoken (ddY) mouse를 이용한 실험결과이었지만 [2], 다른 연구자들은 같은 실험을 했지만 병인성을 확인하지 못하였다는 실험결과들을 잇달아 발표하였다 [5-7]. 이처럼 재현성이 없는 실험실 연구 결과로 인하여 쿠도아충이 식중독을 일으키는가에 대한 인과성 논란이 현재 진행 중에 있다 [8]. 이에 더하여, 2015년 발생되었다는 11건 사례에 대하여도 쿠도아에 의한 식중독이 아닐 가능성이 제기 되었다. 첫째, 잠복기, 임상증상 등의 역학적 특성을 살펴보았을 때 Staphylococcus aureus (황색포도알균) 혹은 Bacillus cereus (세레우스)에 의한 식중독과 혼재된 것일 수 있다는 것이다. 특히 이들 두 종류의 식중독 모두 역학조사시 독소 및 병원체 검출이 어렵다는 점 때문이다 [9]. 둘째, 11건의 사례 발생의 월별 분포에 대한 차이이다. 일본의 쿠도아 식중독 발생의 약 75%가 8월에서 11월 사이에 발생했다고 보고한 반면 [3], 한국은 8-11월에 총 4건의 사례가 발생하여 일본의 절반 수준인 36.3%이기 때문이다 [4]. 이에 더하여 한국은 5월 한달 동안에만 4건이 발생하였고, 6월과 7월에는 발생 사례가 전무하다는 점에서 질병관리본부의 쿠도아 식중독 사례

정의에 대한 타당성에 의문을 갖게 한다.

그런데 발생사례가 월별로 차이가 난다는 점에 있어 ‘제주산’ 광어회 판매량의 월별 변동과의 관련성을 고려할 필요가 있다 [9]. 다시 말해서, 만약 칠성 쿠도아충이 급성 식중독을 야기하는 병인 원충이라면, ‘제주산’ 광어 섭취를 많이 할수록 사례 발생의 위험 또한 증가할 것이기 때문이다. 이에, 질병관리본부가 보고한 2015년도 쿠도아 식중독 발생 사례의 월별 분포와, 같은 기간에 제주도 수산협동조합 (http://www.jaf-suhyup.co.kr/, 이하 제주수협)이 국내 시장에 제공한 출하량 간의 상관성 (correlation)을 알아보는 것이 본 연구의 목적이다. 본 생태학적 연구 (ecologic study)를 통해 쿠도아 충이 급성 식중독의 병인 원충이라는 가설에 대한 인과성 평가의 근거를 추가할 것을 기대한다.

연구대상 및 방법

제주수협의 협조를 받아, 제주도 광어의 2014년 3월부터 2016년 2월까지의 월별 국내 출하량 자료를 확보하였다 (Appendix 1). 사계절 출하량의 추세를 살펴보기 위하여 3-5월, 6-8월, 9-11월, 12월-익년 2월로 재분류하였다.

쿠도아발생 사례에 관한 정보는 질병관리본부가 2016년도에 발표한 2015년도 쿠도아 식중독 사례 보고서 (case reports)이다 [4]. 이는 국내 사례를 질본이 처음으로 발표한 것으로, 2015년 한 해동안 전국에서 신고받은 것에서 분류한 사례로 해석하고, 11건의 사례를 월별로 정리하였다.

2015년도 월별 출하량에 대하여 같은 시기별 쿠도아 식중독 발생 사례 건수와의 상관성 유무를 알아보기 위하여, 비모수검정법인 Spearman's rho와 Kendall's tau-a 계수를 산출하였으며, 유의수준 0.05를 기준으로 상관성 여부를 평가하였다. 출하량과 사례 발생간의 시간적 지연 정도를 알아보기 위하여 시계열 분석의 하나인 교차상관분석 (cross correlation)을 하였고 12개월 자료임을 감안하여 지연을 3개월로 정하였다. 이상의 분석을 위하여 STATA (www.stata.com) version 14를 사용하였다.

연구결과

2014-2015년도 제주수협에서 출하량을 계절별로 살펴보았을 때, 겨울 – 가을 – 봄 – 여름 순서로 출하 변동이 있었다 (Fig. 1). 2015년도의 월별 출하량과 사례 발생월을 같이 살펴보았을 때, 가장 많은 출하량을 보였던 12월에 사례 발생이 전무하였으며, 출하량이 가장 적은 11월에 1건의 사례가 발생했다 (Fig 2). 또한 5월은 5번째로 출하량이 많았지만 가장 많은 사례 발생이 있었다. 출하량과 사례건수에 대한 비모수 상관분석 모두 p-값이 0.05 이상으로 서로 독립적인 것으로 나왔다 (Spearman's rho = 0.39, p-value=0.21; Kendall's tau-a =0.27, p-value =0.20). 추가로 교차상관분석에서 월별 기간 지연에 따른 상관성은 없는 것으로 나왔다 (Tab 1).

토론

2015년도 제주도 양식 광어의 월별 출하량과 같은 연월의 쿠도아 식중독 사례 발생 건과는 상호 독립적이라는 것을 확인하였다. 이상의 생태학적 연구 결과는 두 가지 의미를 갖는다. 첫째, 질병관리본부가 정의한 쿠도아 식중독 사례 정의를 재고할 필요가 있다는 점이다. 둘째, 현 시점에서 사례 정의를 수용한다면, 쿠도아충이 광어의 기생충일 뿐 사람에 있어 식중독을 일으키는 병인 원충이 아니라는 주장을 지지하는 근거가 된다.

사례 발생이 월별로 차이가 나는 것에 대하여 질병관리본부는 해수온도의 차이에 따라 검출율이 달라진다고 해석하고 있다 [4]. 그러나 해수온도와 무관하게 연중으로 칠성 쿠도아충이 검출된다는 주장과 함께 [10], 쿠도아충은 자연산에서는 발견되지 않는다는 보고 [11]를 감안할 때, 해수 온도의 차이로 발생의 계절성 차이를 설명하기에는 설득력이 낮다.

한편 일본은 여름과 가을에 사례 발생이 집중하는 반면 [3], 한국은 여름인 7,8월에 발생 사례가 전무하였고, 5월인 봄에 발생이 가장 많다는 점에서 [4], 수온의 변화보다는 광어회 취식 기회가 월별로 차이난 것에 기인한 것으로 해석하려는 노력이 더 합당할 것이다. 본 연구결과는 출하량 변동도 계절 차이를 설명하지 못한다는 결론을 얻어내었다.

본 연구는 다음의 4가지 주요 한계점을 갖는다.

첫째, 분석에 사용한 광어 출하량이 실제 소비량과 같은 의미를 갖는가에 대한 지적이다. 그런데 횟감으로 사용하는 양식 광어는 최종 소비자까지 활어로 제공되어야 하므로, 주문량에 맞추어 출하량이 이루어진다. 따라서 출하량과 소비량이 일치하지는 않지만 정비례 관계가 있다고 볼 수 있어, 상관 분석으로 이를 극복하였다.

둘째, 출하 시점과 섭취 시점 간에 간격이 있다면, 상관성이 없게 나올 수 있다는 지적이다. 그러나 광어는 주로 생 회로 먹는다는 점에서, 횟집에서는 가능한 신선한 상태로 제공할 수 있도록 가급적 빨리 소비한다. 그 시간 간격이 며칠은 가능하지만 월 단위로 넘어가기는 어려울 것이며, 본 연구에서는 일 단위가 아닌 월 단위로 분석하였고, 교차상관분석에서 지연기간에 따른 변동이 없음을 확인하였다.

셋째, 질본의 보고서 내용에서 쿠도아 식중독의 발생 사례 건 (number of incidents)를 이용하였으며, 각 사례별 환자수 (number of patients)를 사용하지 않았다는 점이다. 쿠도아 식중독은 공통단일원인에 의한 식중독 집단 발생으로 [4], 한 사례별로 환자수가 매우 다양하다. 따라서 생태학적 분석을 위해서는 환자 수보다는 발생 사례 건수를 활용하는 것이 더 타당하다고 보았다.

넷째, 연구 설계상 생태학적 분석으로 개별 자료가 아닌 집단 자료를 사용하여 생기는 생태학적 오류 (ecologic fallacy)가 개입되는 점이다 [12]. 따라서 인과성에 대한 규명

주장보다는 새로운 가설을 제시하는 수준에서 연구결과를 추론해야 한다 [13]. 본 연구 결과는 쿠도아충이 급성 식중독을 일으키는 병인 원충이란 기존의 주장들에 있어, 보다 타당한 인과성 여부를 규명하는 역학 연구가 필요하다는 점을 제기하고자 한다.

Acknowledgement

출하량 자료 제공에 협조해 주신 제주 어류양식수산업협동조합의 김성미 팀장님께 감사를 드립니다.

REFERENCES

1. Matsukane Y, Sato H, Tanaka S, Kamata Y, Sugita-Konishi Y. *Kudoa septempunctata* n. sp. (Myxosporea: Multivalvulida) from an aquacultured olive flounder (*Paralichthys olivaceus*) imported from Korea. Parasitol Res 2010;107:865-72.

2. Kawai T, Sekizuka T, Yahata Y, Kuroda M, Kumeda Y, Iijima Y, et al. Identification of *Kudoa septempunctata* as the causative agent of novel food poisoning outbreaks in Japan by consumption of *Paralichthys olivaceus* in raw fish. Clin Infect Dis 2012;54:1046-52.

3. Sugita-Konishi Y, Sato H, Ohnishi T. Novel foodborne disease associated with consumption of raq fish, olive flounder (*Paralichthys olivaceus*). Food Safety 2014;2:141-50.

4. Centers for Disease Control and Prevention in Korea. Case reports of Kudoa food poisoning in 2015; 2016 [cited 2017 Aug 11]. Available from: http://cdc.go.kr/CDC/notice/CdcKrTogether0302.jsp?menuIds=HOME001-MNU1154-MNU0005-MNU0088&cid=71877 (Korean).

5. Guo Q, Jia L, Qin J, Li H, Gu Z. Myxozoans and Our Dinner Table: Pathogenicity Studies of *Myxobolus honghuensis* (Myxosporea: Bivalvulida) Using a Suckling Mice Model. Foodborne Pathog Dis 2015;12:653-60.

6. Ahn M, Woo H, Kang B, Jang Y, Shin T. Effect of oral administration of *Kudoa septempunctata* genotype ST3 in adult BALB/c mice. Parasite 2015;22:35.

7. Jang Y, Ahn M, Bang H, Kang B. Effects of *Kudoa septempunctata* genotype ST3 isolate from Korea on ddY suckling mice. Parasite 2016;23:18.

8. Chung YB, Bae JM. Is there evidence that *Kudoa septempunctata* can cause an outbreak of acute food poisoning? Epidemiol Health 2017;39:e2017004.

9. Lee SU. Analysis of *Kudoa septempunctata* as a cause of foodborne illness and its associated differential diagnosis. Epidemiol Health 2017;39:e2017014.

10. Ohnishi T, Furusawa H, Sako H, Ototake M, Fukuda Y, Yoshinari T, et al. Studies on seasonal changes in occurrence of food-borne disease associated with *Kudoa septempunctata*. Jpn J Food Microbiol 2013;30:125-131 (japanese).

11. Song JY, Choi JH, CHoi HS, Jung SH, Park MA. Monitoring of *Kudoa septempunctata* in cultured olive flounder and wild fish in Jeju island during 2012. J Fish Pathol 2013;26:129-137.

12. Wakefield J. Ecologic studies revisited. Annu Rev Public Health 2008;29:75-90.

13. Lee SU. Analysis of Kudoa septempunctata as a cause of foodborne illness and its associated differential diagnosis. Epidemiol Health 2017;39:e2017037r.

Table 1. Cross-correlation coefficients by time lags

Lags (month)	Cross correlation coefficient
-3	0.000
-2	0.077
-1	0.000
0	0.237
1	0.000
2	-0.007
3	0.000

<Appendix 1> Shipping amounts of olive flounder from Jejudo from January 2014 to February 2016.

Time	Amount (ton)
2014_Jan	1,858
2014_Feb	2,038
2014_Mar	2,262
2014_Apr	2,143
2014_May	2,225
2014_Jun	2,197
2014_July	2,066
2014_Aug	1,922
2014_Sep	2,099
2014_Oct	2,564
2014_Nov	2,002
2014_Dec	2,906
2015_Jan	2,443
2015_Feb	2,403
2015_Mar	1,986
2015_Apr	2,220
2015_May	2,335
2015_Jun	2,000
2015_July	2,013
2015_Aug	2,199
2015_Sep	2,531
2015_Oct	2,214
2015_Nov	1,988
2015_Dec	2,809

2016_Jan	2,290
2016_Feb	2,371
